# Effect of Inoculation Level on the Impact of the PGPR *Azospirillum lipoferum* CRT1 on Selected Microbial Functional Groups in the Rhizosphere of Field Maize

**DOI:** 10.3390/microorganisms10020325

**Published:** 2022-01-31

**Authors:** Sébastien Renoud, Danis Abrouk, Claire Prigent-Combaret, Florence Wisniewski-Dyé, Laurent Legendre, Yvan Moënne-Loccoz, Daniel Muller

**Affiliations:** 1 Univ Lyon, Université Claude Bernard Lyon 1, CNRS, INRAe, VetAgro Sup, UMR5557 Ecologie Microbienne, 43 bd du 11 Novembre 1918, F-69622 Villeurbanne, France; seb.renoud@gmail.com (S.R.); danis.abrouk@univ-lyon1.fr (D.A.); claire.prigent-combaret@univ-lyon1.fr (C.P.-C.); Florence.Wisniewski@univ-lyon1.fr (F.W.-D.); laurent.legendre@univ-lyon1.fr (L.L.); yvan.moenne-loccoz@univ-lyon1.fr (Y.M.-L.); 2 Département de Biologie Biochimie, Univ Lyon, Université Jean Monnet, UFR des Sciences et Techniques, F-42000 Saint-Etienne, France

**Keywords:** functional community, diazotrophs, nitrogen fixers, ACC deaminase producers

## Abstract

The impact of inoculated plant growth-promoting rhizobacteria (PGPR) on its host physiology and nutrition depends on inoculum level. Whether the impact of the inoculated PGPR on the indigenous rhizosphere microbiota also varies with the PGPR inoculum level is unclear. Here, we tested this issue using the PGPR *Azospirillum lipoferum* CRT1—maize model system, where the initial seed inoculation is known to enhance maize growth and germination, and impacts the maize rhizomicrobiota, including microbial functional groups modulating plant growth. *A. lipoferum* CRT1 was added to the seeds at standard (10^5–6^ cells.seed^−1^) or reduced (10^4–5^ cells.seed^−1^) inoculation levels, in three fields. The effect of the two PGPR formulations was assessed on maize growth and on the *nifH* (nitrogen fixation), *acdS* (ACC deaminase activity) and *phlD* (2,4-diacetylphloroglucinol production) microbial functional groups. The size of the three functional groups was monitored by qPCR at the six-leaf stage and the flowering stage, and the diversity of the *nifH* and *acdS* functional groups (as well as the bacterial community) were estimated by MiSeq metabarcoding at the six-leaf stage. The results showed that the benefits of the reduced inoculant formulation were significant in two out of three fields, but different (often lower) than those of the standard formulation. The effects of formulations on the size of the three functional groups differed, and depended on field site and functional group. The reduced formulation had an impact on the diversity of *nifH* and *acdS* groups at one site, whereas the standard formulation had an impact at the two other sites. Inoculation significantly impacted the total bacterial community in the three fields, but only with the reduced formulation. In conclusion, the reduced inoculant formulation impacted the indigenous rhizosphere microbiota differently, but not less efficiently, than the standard formulation.

## 1. Introduction

The inoculation of plants with beneficial microorganisms such as Plant-Growth-Promoting Rhizobacteria (PGPR) to enhance crop productivity is an over one-hundred-year- old farming technique [1,2]. Indeed, PGPR (encompassing a diversity of bacterial taxa [3]) can benefit plant growth by several mechanisms, such as (i) modulating plant hormonal balance via the production of auxins, gibberellins, abscisic acid or 1-aminocyclopropane-1-carboxylate (ACC) deaminase, resulting in a stimulation of root-system development and soil prospection by roots, and (ii) the improvement of plant nutrition by phosphate solubilisation or atmospheric nitrogen fixation [4,5,6,7,8]. In Latin America, South-East Asia and Africa, inoculated seeds are sown at a large scale, with millions of hectares of *Fabaceae* (e.g., soybean or bean) and *Poaceae* (e.g., maize, sorghum or wheat) inoculated by PGPR belonging mainly to the genera *Bacillus, Paenibacillus*, *Pseudomonas* or *Azospirillum* [2,9,10,11,12]. In the context of ecological awareness in agronomy, biofertilisation with microorganisms constitutes an attractive alternative to chemical inputs, which are polluting and decrease soil fertility [13,14,15,16]. Various studies reported that PGPR inoculant functioning under field conditions is impacted by soil type and plant genotype, as well as the PGPR strains used [2,9,10,17,18,19,20,21].

Unlike under greenhouse conditions, field-inoculation of phytobeneficial microorganisms is associated with variable levels of success. Consequently, the development of inoculant formulation (i.e., the combination of the bacterial strain with a carrier) that provides more suitable micro-environmental conditions can be useful to improve inoculant performance [2,22]. The inoculant formulation should stimulate the optimum establishment, and functioning, of the inoculant, so as to maximize its effects on the plant. A major objective of the formulation is to deliver a number of inoculant cells sufficient to induce plant stimulation [1,22,23]. Indeed, the inoculum dose has been described as an important factor to observe a beneficial effect on the plant. Generally, the number of inoculant cells necessary should be in the order of 10^6^ to 10^7^ per seeds [24,25] and, in most studied cases, the correlation between inoculum density and phytostimulation effectiveness is tight [26,27,28].

*Azospirillum lipoferum* CRT1 is a natural PGPR isolate that is commercially used on maize to stimulate plant growth, and field yield, with a peat-based seed-coating formulation providing 10^5–6^ CFU of bacteria per seed [29,30]. Nevertheless, phytostimulation can also be achieved at lower inoculation levels, and we evidenced positive effects of *A. lipoferum* CRT1 on maize physiology with only 10^4–5^ inoculant cells per seed [20,21,31]. In addition to direct effects on maize physiology [29], inoculation of maize seeds with *A. lipoferum* CRT1 affects the bacterial community of the rhizosphere [21,32]; this includes specific microbial functional groups important for plant growth [21], such as diazotrophs (nitrogen fixation), ACC deaminase producers (regulators of ethylene metabolism in roots), and producers of 2,4-diacetylphloroglucinol, a root-branching signal with auxinic effects [33]. Therefore, it is likely that phytostimulation by this PGPR involves a combination of direct effects on the plants, as well as indirect effects on the indigenous rhizosphere microbial community. In light of this background, the impact of low inoculant doses should also be evaluated on these indigenous microbial phytostimulators. In particular, we hypothesize that reduced inoculum levels affect, albeit to a lesser efficiency, microbial functional groups in parallel to their effects on maize physiology.

The objective of this work was to assess the effect of a reduced inoculum level (10^4–5^ cells.seed^−1^; formulation F1) of *A. lipoferum* CRT1 on its host physiology and rhizosphere microbial communities, in comparison with a standard inoculum level (10^5–6^ cells.seed^−1^; formulation F2) under agronomic conditions. These maize inoculation experiments were carried out in parallel at three different field sites; additionally, results obtained with the F1 formulation on maize photosynthesis efficiency, metabolome, growth and yield have been previously reported [20], along with findings on nitrifying and denitrifying communities in bulk soil [31], as well as diazotroph communities, ACC deaminase producer communities and 2,4-diacetylphloroglucinol producer communities in the rhizosphere [21]. However, formulation F1 data-enabling to compare the two inoculation levels has not been reported, and this was the focus of this study. Thus, the influence of the inoculation level of *A. lipoferum* CRT1 on the inoculant effect on shoot and root development was investigated. In addition, to consider the possibility of indirect plant-beneficial effects, we also monitored the indigenous rhizosphere community via the size and/or diversity of the diazotrophs, ACC deaminase producers and 2,4-diacetylphloroglucinol producers, and the assessment of the diversity of the overall bacterial community.

## 2. Results

### 2.1. Inoculum Survival

Quantitative PCR analysis of *A. lipoferum* CRT1 DNA on maize rhizosphere soil samples at the six-leaf and flowering stages did not yield any signal, indicating that the inoculant *A. lipoferum* CRT1 was under the detection threshold (i.e., below 4.0 × 10^3^ cell equivalents per g of rhizosphere soil) in the three field trials, irrespective of the formulation. This also indicates that indigenous *Azospirillum* strains closely related to the inoculant were not present in the three field sites.

### 2.2. Effects of Inoculum Level on Maize Development

In the absence of inoculation, the F1 seed mock formulation resulted, at the six-leaf stage, in a 48% decrease in the number of maize roots (at site L), a 10% increase in leaf length and a 10% increase in average root diameter (at site FC), a 4% decrease in total root length (at site FC), and a 9% increase in leaf width (at site C) in comparison with the F2 mock (control) treatment (Table 1).

At site L, seed inoculation with *A. lipoferum* CRT1 resulted in significantly higher shoot biomass (+26% with F1 vs. +29% with F2), leaf width (+10% with F1 only) or leaf length (+10% with F2 only), stem diameter (+11% with F1 vs. +13% with F2), root biomass (+20% with F1 only), total root length (+43% with F1 vs. +47% with F2), total root surface (+42% with F1 vs. 24% with F2) and number of roots (+91% with F1 vs. +160% with F2), but lower average root diameter (−14% with F2 only), in comparison with the corresponding non-inoculated mock controls (Table 1). At site FC, inoculation caused an 18% decrease in root biomass with F1 only (compared with the F1 control), whereas with F2 it led to higher shoot biomass (+26%), leaf length (+10%), stem diameter (+12%) and number of roots (+29%) in comparison with the F2 non-inoculated control (Table 1). At site C, the use of CRT1 resulted in higher shoot biomass (+15% with F1 vs. +24% with F2), leaf length (+9% with F2 only), leaf width (+12% with F2 only) and stem diameter (+10% with F2 only), but lower average root diameter (−9% with F1 vs. −10% with F2), compared with the corresponding non-inoculated mock controls (Table 1).

### 2.3. Effects of Inoculum Level on Numbers of nifH, acdS and phlD Rhizobacteria

**Figure 1:**
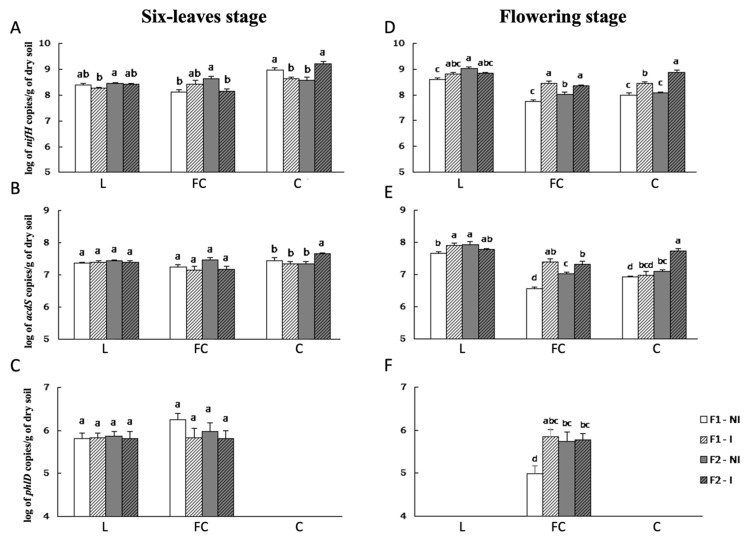
Effect of inoculation level of *A. lipoferum* CRT1 on the size of *nifH* (**A**,**D**)*, acdS* (**B**,**E**) and *phlD* (**C**,**F**) functional groups at two sampling times of maize in three fields. L, FC and C correspond to the three sites studied. Data are shown in log base 10, with standard errors (*n* = 20). F1 corresponds to the peat matrix used to coat seeds with low inoculant concentration, and the data originate from [21]. In F2, *A. lipoferum* CRT1 was used at higher inoculant concentration. NI and I correspond, respectively, to non-inoculated and inoculated conditions. In C and F, some of the samples were below the detection threshold (3.2 × 10^3^ *phlD* copies per gram of lyophilized soil). At each field site, statistical differences between the four treatments are indicated by letters a–d (ANOVA and Fischer’s LSD tests, *p* < 0.05).

In the absence of the inoculant bacterium, seed mock-coating had an effect on the size of the three functional groups under scrutiny at certain field sites and sampling dates. At the six-leaves stage, the number of rhizosphere *nifH* bacteria in the F1 control samples was lower in FC (−0.5 log) but higher in C (+0.4 log) than in the F2 control samples. At the flowering stage, the F1 control rhizosphere soil samples had a reduced *nifH* group in L (−0.4 log) and FC (−0.3 log), *acdS* group in L (−0.3 log), FC (−0.5 log) and C (−0.2 log), and *phlD* group in FC (−0.7 log), in comparison with the F2 control (Figure 1).

At the six-leaf stage, seed inoculation with *A. lipoferum* CRT1 resulted in a significantly different size for the *nifH* group at sites L (+0.7 log with F2 only), FC (−0.5 log with F2 only) and C (−0.3 log with F1 only) and for the *acdS* group in C (+0.3 log with F2 only), but not for the *phlD* group (Figure 1). At the flowering stage, *A. lipoferum* CRT1 inoculation increased the size of the *nifH* group in FC (+0.7 log with F1 vs. +0.3 log with F2) and C (+0.5 log with F1 vs. +0.8 log with F2), of the *acdS* group in L (+0.2 log with F1 only), FC (+0.8 log with F1 vs. +0.3 log with F2) and C (+0.6 log with F2 only), and of the *phlD* group in FC (+0.9 log with F1 only).

### 2.4. Effects of Inoculum Level on nifH and acdS Diversity of Maize Rhizosphere

NMDS evidenced that the main differences in the *nifH*, *acdS* and *rrs* datasets of the six-leaf-maize rhizosphere soil were due to field site peculiarities (Figures S7, S8 and S9, respectively). To unmask the overriding effects of field conditions, the impact of inoculation was assessed by BCA.

**Figure 2:**
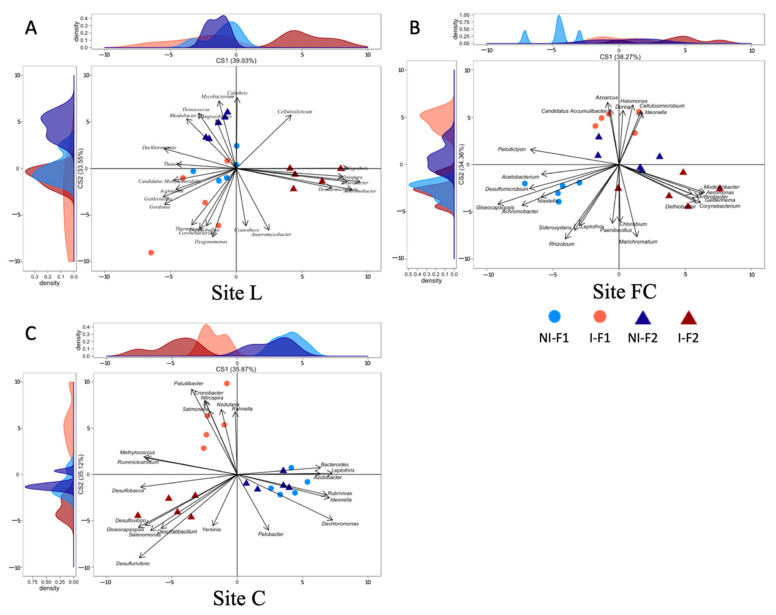
Effect of inoculation level of *A. lipoferum* CRT1 on the composition of the diazotroph community (*nifH*) as indicated by between-class analyses (BCA) studied at three field sites L (**A**), FC (**B**), and C (**C**). F1 (circles) corresponds to the peat matrix used to coat seeds with low inoculant concentration, using data originating from [21], and in F2 (triangles), *A. lipoferum* CRT1 was used at higher inoculant concentration. NI (blue) and I (reddish) correspond, respectively, to non-inoculated and inoculated conditions. Curves on top and left represent, respectively, the sample distribution on abscissa and ordinate axes. Vectors on plot represent the bacterial genera contributing the most to sample distribution.

**Figure 3:**
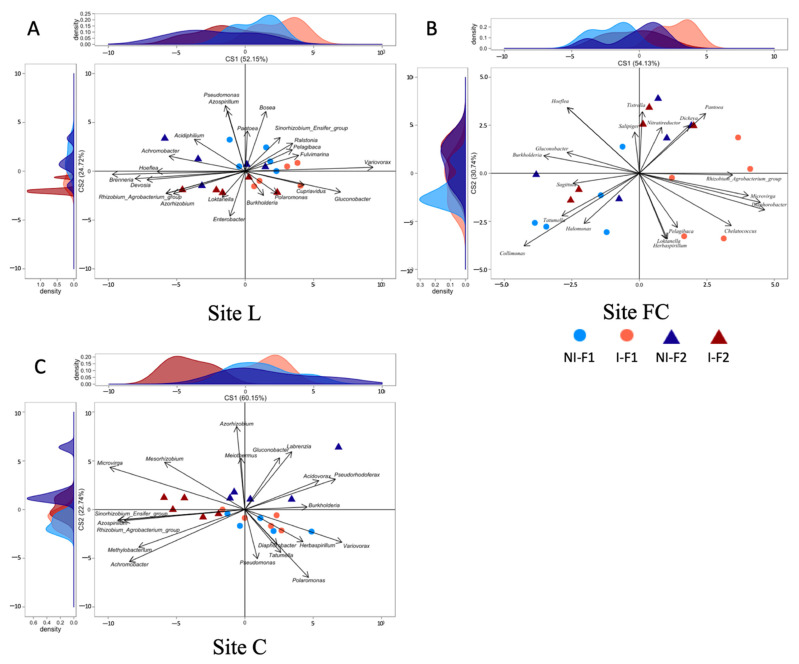
Effect of inoculation level of *A. lipoferum* CRT1 on the composition of the ACC deaminase producers (*acdS*) as indicated by between-class analyses (BCA) studied at three field sites L (**A**), FC (**B**), and C (**C**). F1 (circles) corresponds to the peat matrix used to coat seeds with low inoculant concentration, using data originating from [21], and in F2 (triangles), *A. lipoferum* CRT1 was used at higher inoculant concentration. NI (blue) and I (reddish) correspond, respectively, to non-inoculated and inoculated conditions. Curves on top and left represent, respectively, the sample distribution on abscissa and ordinate axes. Vectors on plot represent the bacterial genera contributing the most to sample distribution.

First, we compared the two treatments with non-bacterized formulations. BCA on the *nifH* group at sites L, FC and C revealed no difference (*p* > 0.05) in taxa composition (Figure 2). For the *acdS* group, there was no difference at sites L (*p* = 0.29 for axis X and 0.08 for axis Y) and FC (*p* = 0.60 for axis X and 0.18 for axis Y), whereas at C, the F1 and F2 control samples differed along the ordinate axis (*p* = 0.004; 24% of between-class variability), especially for the genera *Brevibacterium, Micromonospora*, *Lechevalieria*, *Phycicoccus*, *Rhodococcus* and *Mycobacterium* (genera most commonly associated with the non-inoculated formulation F1) and *Azorhizobium*, *Mesorhizobium*, *Meiothermus*, *Microvirga*, *Labrenzia*, and *Nesterenkonia* (genera most commonly associated with the non-inoculated formulation F2) (Figure 3).

Second, we compared the *nifH* group in the rhizosphere soil of non-inoculated and *A. lipoferum* CRT1-inoculated maize (Figure 2). Based on the BCA of *nifH* data, the effect of bacterial inoculation at site L was not significant with F1 (*p* = 0.54 for axis X and 0.54 for axis Y) but was significant with F2 along the abscissa axis (*p* = 0.004; 39% of between-class variability), where the most discriminant genera were *Geitlerinema, Dechloromonas, Gordonia, Candidatus Methylomirabilis, Acetonema* and *Thauera* (most commonly associated with non-inoculated maize) and *Nostoc, Achromobactin, Tolypothrix, Arthrobacter, Salinispora* and *Desulfomicrobium* (genera most commonly associated with inoculated maize); bacterial inoculation was also significant with F2 along the ordinate axis (*p* = 0.004; 34% of between-class variability), where the most discriminant genera were *Calothrix, Mycobacterium, Deinococcus, Cellulosilyticum, Rhodobacter* and *Mangrovibacter* (most commonly associated with non-inoculated maize) and *Dysgonomonas, Anaeromyxobacter, Corynebacterium, Cyanothece, Desulfobulbus* and *Thermincola* (genera most commonly associated with inoculated maize). At FC, inoculation had an effect with F1 along the ordinate axis (*p* = 0.007; 34% of between-class variability), with *Rhizobium, Marichromatium, Sideroxydans, Leptothrix, Paenibacillus* and *Chlorobium*, most commonly associated with non-inoculated maize, and *Azoarcus, Halomonas, Candidatus Accumulibacter, Dorea, Cellulosimicrobium* and *Ideonella* genera most commonly associated with inoculated maize, but not with F2 (*p* = 0.54 for axis X and 0.14 for axis Y). At site C, inoculation had no effect with F1 (*p* = 0.09 for axis X and 0.14 for axis Y), but F2 samples differed from mock samples along the abscissa axis (*p* = 0.001; 36% of between-class variability). The most discriminant genera were *Dechloromonas, Leptothrix, Ideonella, Rubrivivax, Bacteroides* and *Azotobacter* (genera most commonly associated with non-inoculated maize) and *Gloeocapsopsis, Desulfurivibrio, Desulfobacca, Methylococcus, Desulfovibrio* and *Ruminiclostridium* (genera most commonly associated with inoculated maize).

Third, we assessed the *acdS* group in non-inoculated and inoculated maize rhizosphere soil samples (Figure 3). Based on the BCA of *acdS* data, there was no effect of inoculation at site L with F1 (*p* = 0.90 for axis X and 0.07 for axis Y), but there was a difference with F2 along the ordinate axis (*p* = 0.038; 24% of between-class variability). The most discriminant taxa were *Bradyrhizobium, Chelatococcus, Nesterenkonia, Herbaspirillum*, *Halomonas* and *Pelagibaca*, most commonly associated with non-inoculated maize, and *Nocardia, Collimonas, Gluconobacter, Komagataeibacter* and *Diaphorobacter*, most commonly associated with inoculated maize. At FC, F1 inoculation had an effect along the abscissa axis (*p* = 0.017; 53% of between-class variability), and the most discriminant genera were *Collimonas, Nesterenkonia, Burkholderia, Tatumella, Pseudonocardia* and *Phycicoccus* (genera most commonly associated with non-inoculated maize), and *Tetrasphaera, Modestobacter, Serinicoccus, Roseovarius, Mesorhizobium* and *Actinoplanes* (genera most commonly associated with inoculated maize), but no significant difference was observed between non-inoculated and inoculated maize with F2. At C, the effect of inoculation was not significant with F1 (*p* = 0.99 for axis X and 0.85 for axis Y) but was significant with F2 along the abscissa axis (*p* = 0.030; 55% of between-class variability), and the most discriminant genera were *Starkeya, Streptomyces, Achromobacter, Rhizobium/Agrobacterium, Sinorhizobium/Ensifer* and *Microvirga* (genera most commonly associated with non-inoculated maize), and *Variovorax, Pseudorhodoferax, Acidovorax* and *Methylibium* (genera most commonly associated with inoculated maize).

In summary, seed inoculation with *A. lipoferum* CRT1 resulted in significantly different compositions of the *nifH* and *acdS* groups at each field site, but this depended on the inoculation level. For both functional groups, the bacterial genera contributing most to the distinction between inoculated and uninoculated maize samples were different between fields. Moreover, within a field, their individual contribution depended on the level of CRT1 inoculation, indicating field-specific effects as well as formulation-specific effects on native rhizosphere bacteria in these functional groups.

### 2.5. Effects of Inoculum Level on the Genus Composition of the nifH and acdS Groups within the Maize Rhizosphere

Further insight into the effects of CRT1 inoculum level on the *nifH* and *acdS* functional groups were sought by considering the 20 most prevalent genera within these groups, even though differences in their relative abundances were not significant at *p* < 0.05. Among the diazotrophs (Figure S4), there were trends for *Burkholderia, Paenibacillus*, *Clostridum* (at sites L and FC), *Desulfovibrio* (at sites FC and C), *Azospirillum, Bradyrhizobium*, *Skermanella* (at site FC), *Azoarcus*, *Cellulosilyticum*, *Paludibacter* and *Ruminiclostridium* (at site L) to be more prevalent in F1 inoculated maize. There were also trends for *Geobacter* (at sites L and FC), *Bradyrhizobium* (at sites C and L), *Paenibacillus*, *Pelosinus, Pseudomonas* (at sites C), *Dechloromonas, Desulfovibrio, Rhizobium, Skermanella* (at sites L) and *Azoarcus* (at site FC) to be more prevalent in the rhizophere of F2-inoculated maize than in F1-inoculated maize.

Among the ACC deaminase producers, there were trends for *Methylibium, Polaromonas* (at sites FC and C), *Bradyrhizobium* (site FC), *Pseudomonas* and *Variovorax* (site C) to be more prevalent after maize F1 inoculation (Figure S5). In F2-inoculated maize rhizosphere samples, there were trends for *Burkholderia* (at the three sites), *Rhizobium* (at sites C and L), *Pseudomonas*, *Variovorax* (site FC), *Bradyrhizobium* and *Mesorhizobium* (site C) to be more prevalent. Thus, for both functional groups, contrasting trends were observed for the majority of these genera as a function of field site, but also of inoculant formulation.

### 2.6. Effects of Inoculum Level on the Total Bacterial Diversity of Maize Rhizosphere

**Figure 4:**
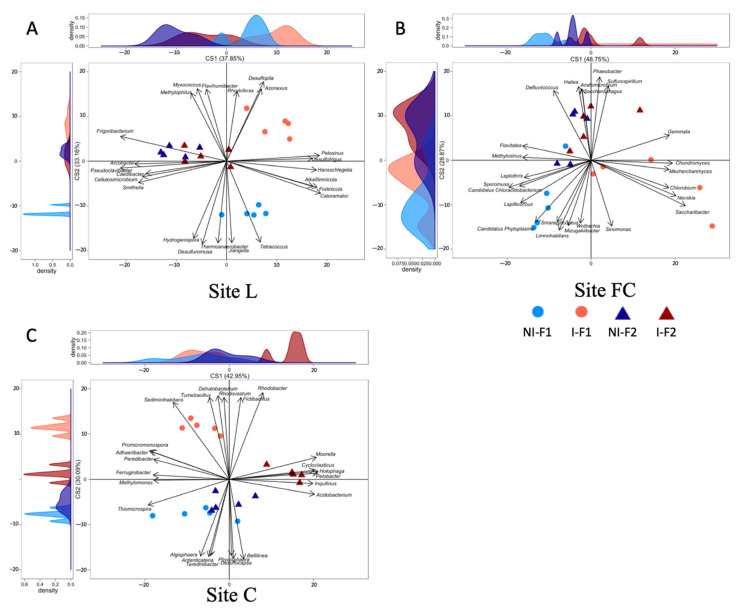
Effect of inoculation level of *A. lipoferum* CRT1 on the composition of the total bacterial community (*rrs*) as indicated by between-class analyses (BCA) studied at three field sites L (**A**), FC (**B**), and C (**C**). F1 (circles) corresponds to the peat matrix used to coat seeds with low inoculant concentration, using data originating from [21], and in F2 (triangles), *A. lipoferum* CRT1 was used at higher inoculant concentration. NI (blue) and I (reddish) correspond, respectively, to non-inoculated and inoculated conditions. Curves on top and left represent, respectively, the sample distribution on abscissa and ordinate axes. Vectors on plot represent the bacterial genera contributing the most to sample distribution.

First, we compared the F1 and F2 non-inoculated control treatments on the basis of the taxonomic composition of the rhizosphere bacterial communities, using the BCA (Figure 4) of Illumina MiSeq sequencing of 16S rRNA genes (*rrs*). At site L, the controls differed along the abscissa axis (*p* = 0.044; 38% of between-class variability), with *Caloramator, Pelosinus, Fodinicola, Hansschlegelia, Alkalilimnicola* and *Desulfofrigus* most commonly associated with the F1 mock treatment, and *Pseudoclavibacter, Frigoribacterium, Arcobacter, Smithella, Cellulosimicrobium* and *Caedibacter* most commonly associated with the F2 mock treatment. The effect of formulation type was not significant at sites FC in the absence of bacterial inoculant (*p* = 0.40 for axis X and 0.10 for axis Y) and C (*p* = 0.44 for axis X and 0.67 for axis Y).

Second, the BCA showed that the effect of inoculation was significant at all three sites with F1, i.e., at site L along the ordinate axis (*p* = 3.6 × 10^−3^; 33% of between-class variability), with the most discriminant genera being *Candidatus Metachlamydia, Desulfuromusa, Thermoanaerobacter, Jiangella, Tetracoccus* and *Hydrogenispora* (genera most commonly associated with non-inoculated maize), and *Desulfopila, Myxococcus, Azonexus, Flavihumibacter, Rhodoferax* and *Methylophilus* (genera most commonly associated with inoculated maize); at site FC along the abscissa axis (*p* = 6.9 × 10^−3^; 29% of between-class variability), with *Candidatus Chloracidobacterium, Sporomusa, Lapillicoccus, Methylosinus, Flavitalea* and *Leptothrix* most commonly associated with non-inoculated maize, whereas *Saccharibacter, Nevskia, Chondromyces, Gemmata, Mechercharimyces* and *Chlorobium* were genera most commonly associated with inoculated maize; and at site C along the ordinate axis (*p* = 5.5 × 10^−3^; 29% of between-class variability), with *Bellilinea, Desulfocapsa, Ardenticatena, Algisphaera, Teredinibacter* and *Phycisphaera* being most commonly associated with non-inoculated maize, and *Rhodobacter, Dehalobacterium, Fictibacillus, Tumebacillus*, *Rhodovastum* and *Sediminihabitans* most commonly associated with inoculated maize (Figure 4). In contrast, inoculation with F2 had no significant effect at sites L (*p* = 0.74 for axis X and 0.98 for axis Y), FC (*p* = 0.83 for axis X and 0.92 for axis Y) and C (*p* = 0.32 for axis X and 0.47 for axis Y).

Third, the analysis of taxonomic composition data restricted to the 20 most abundant rhizosphere bacterial genera (Figure S6) did not enable the identification of trends of variations associated with treatment type. However, these genera only accounted for about 40% of all sequences.

## 3. Discussion

In this work, a reduced formulation F1 providing only 10^4–5^ cell equivalents per seed was assessed in comparison with the F2 standard inoculum level of 10^5–6^ cell equivalents [34]. This reduced inoculum level did not lead to enhanced maize growth at field FC (unlike the standard formulation F2), but improved growth in the other fields (less than F2 at site C, but to a similar extent at site L). *Azospirillum* inoculation with the F1 formulation resulted in diminished grain yield at site FC (although it had increased yield in the same field the year before), gave a positive trend (not significant at *p* < 0.05) with a 16% yield increase at site C, and enhanced yield significantly at site L based on data in Rozier et al. [20]. In comparison, inoculation with the F2 formulation did not improve yield [20], which means that reducing the inoculum level was, in fact, a positive approach in terms of maize phytostimulation.

qPCR assessment of inoculant levels during plant growth in the field showed that the inoculant had failed to survive durably in the rhizosphere [21], which was also found in this work when using the higher F2 inoculum level. This reinforces the concept that *A. lipoferum* CRT1 acts mainly during the very early stages of maize development, i.e., in the spermosphere [20,29,35,36]. Besides enhanced growth, significant effects had been observed under these conditions on photosynthesis, and on leaf and root metabolite contents [20]. *A. lipoferum* CRT1 was also found, in other maize experiments, to (i) stimulate early root radicle emergence [20,36], (ii) modify the chemical structure of root cell walls, which might improve water retention and resistance to mechanical stress [37], and (iii) affect root and shoot contents of seedlings in phenolic secondary metabolites [38,39].

The quantity of peat-based coating used to associate the inoculated bacteria to the seeds positively affected maize growth. We observed better growth with the non-bacterized F2 control treatment that implicated higher levels of peat-based coating than the F1 control. Soil amendment with peat can influence plant growth parameters and microbiota activities (e.g., cellobiosidase and ß-xylosidase activities; [40]), but this was seen with high amounts of peat (300 m^3^ ha^−1^). Here, less than 10 dm^3^ of peat was used per ha, so the corresponding nutrient addition was negligible. However, peat contains humic acids [41,42], which may act as auxin-like compounds on maize [43] and might account for root system stimulation. The F2 control treatment also enhanced the abundance of the three studied bacterial functional groups, although enhancement levels were modest.

The main focus of this work was the ecological impact of reduced CRT1 inoculation level on resident rhizosphere bacteria, and more specifically, on key microbial functional groups [21]. The absence of detectable inoculant survival made it unlikely that such an impact would entail strong direct effects of CRT1 cells on neighbouring bacteria colonizing root surfaces, particularly at the reduced inoculation level, but might rather be linked to indirect effects mediated through changes in plant traits [20,36]. Indeed, the BCA of *rrs* sequences showed that the composition of the rhizobacterial community differed following inoculation with reduced amounts of *A. lipoferum* CRT1 (i.e., with the F1 formulation [21]) at each of the three field sites, but not with the standard CRT1 inoculation (10^5–6^ CFU per seed; F2 formulation), pointing to effects of a larger magnitude with the F1 formulation.

Nitrogen fixation and ACC deamination are functions harboured by a minority of taxa [44,45]. In the NCBI database, the *nifH*^+^ bacteria account for only 4.5% and the *acdS*^+^ bacteria for 8.0% of all available sequenced bacterial genomes (*n* = 10,369). In Orr et al. [46], analyses of microbiota composition revealed that the *nifH* community accounted for approximatively 2% of the total community based on qPCR assays in the rhizosphere of barley. Sequencing performed in sugarcane [47] and soybean [48] confirmed the small proportion of the diazotroph community. Thus, inoculation effects on these functional communities may not necessarily parallel changes at the level of the total community. Furthermore, the capacity to fix nitrogen (or deaminate ACC) is not shared by all species/strains of a given genus (for a survey see [44]). In *Azospirillum*, *acdS* is harboured by *A. lipoferum* strains 4B and B510, but not by *A. lipoferum* CRT1, *A. baldaniorum* (formerly *A. brasilense*) Sp245 or *A. brasilense* CBG497 [44]. It should also be noted that host plants may select microorganisms with particular function(s) in the rhizosphere, regardless of the taxa they belong to [45,47,48,49,50].

Inoculation effects on the size of rhizosphere bacterial functional groups (when taking place) were of similar magnitude with the two formulations (a 0.2–0.9 log difference). Since inoculation effects on maize growth were more pronounced with F2 (than with F1), one might have expected that F2 inoculation treatments would have led to larger amounts of nutrients exudated by plant roots [51,52] and, in turn, bigger sizes of bacterial functional communities, which was not the case. This suggests that differences in exudate quality might have played a larger role.

Inoculation of *A. lipoferum* CRT1 with formulation F1 or F2 had a field-specific impact on the taxonomic composition of the two functional groups (*nifH* and *acdS*), as the effects on both were significant at site FC with formulation F1 vs. sites L and C with formulation F2. FC was the only site where inoculation using formulation F1 resulted in reduced maize growth, therefore suggesting that the impact of F1-formulated CRT1 on these two functional groups was, in this site, a consequence of the direct inoculant effects on maize, rather than a modification contributing to phytostimulation. Indeed, the quantity and quality of root exudates can correlate with root biomass and, more globally, plant development [51,52,53]. In the two other sites (FC and C), inoculation with formulation F2 showed enhanced impact on root and shoot development when compared with inoculation F1, and this enhanced impact coincided with a modification of the composition of the bacterial functional communities. Distinct effects of CRT1 concentration on the *nifH* vs. *acdS* functional groups were evidenced when considering the main genera involved, as these effects were both field- and formulation-specific. It suggests that the ecology of these genera may be governed differently depending on local abiotic and biotic factors, and that the possibility of predicting the impact of inoculation on a given bacterial genus is probably very limited.

In conclusion, this work showed that a thinner formulation providing a lower inoculant level resulted in similar, more pronounced or less pronounced effects on the size of six-leaf plants (depending on field site) although the effects on yield were always more important. Inoculation with *A. lipoferum* CRT1 affected the size and taxonomic composition of the functional communities involved in nitrogen fixation (*nifH*) or ACC deamination (*acdS*), with formulation-specific and field-specific manners. Therefore, our initial hypothesis that reduced inoculum levels should lead to reduced effects on microbial functional groups did not materialize. In addition, only the reduced formulation had a significant impact on the total bacterial community, and thus, further research is needed to decipher the *Azospirillum*–maize–microbiota interactions underpinning CRT1 phytostimulation.

## 4. Materials and Methods

### 4.1. PGPR Strain and Inoculum Preparation

*A. lipoferum* CRT1 was isolated from the rhizosphere of maize in a French field [54] and was selected as effective inoculant for maize phytostimulation [29,35,36,54]. Inoculum preparation has been described in detail [20]. The maize seeds (*Zea mays* cv. Seiddi; Caussade Semences, Caussade, France) were inoculated with an industrial coating (Agrauxine Company, Lesaffre Plant Care group, Angers, France) consisting of a proprietary peat-based formulation containing live CRT1 cells [20] to reach a standard inoculation level (F2 formulation) or a reduced inoculation level (formulation F1). The non-inoculated controls consisted of seeds coated under the same conditions and with the exact same peat formulation (i.e., an F2 control and a thinner F1 control), but without *A. lipoferum* CRT1. The coated seeds were prepared separately for site C and for sites L and FC, sown days later.

The inoculum preparation aimed originally at adding 10^7^ cells (for F2) and 10^6^ cells (for F1) of *A. lipoferum* CRT1 per seed [20], but the industrial process, and especially the drying phase, lead to subsequent inoculant loss, resulting in 10^5–6^ cells.seed^−1^ and 10^4–5^ cells.seed^−1^, respectively. Inoculum levels were determined by colony counts on nitrogen-free agar containing ammonium chloride (0.2 g L^−1^) and Congo Red [55], and by quantitative PCR (qPCR) using primers specific for strain CRT1 [34]. At sowing, *A. lipoferum* CRT1 was recovered from seeds at 3.7 × 10^2^ CFU (on plates) and 3.0 × 10^4^ cell equivalents (by qPCR) per seed at sites L and FC, and 8.8 × 10^2^ CFU (on plates) and 1.5 × 10^5^ cell equivalents (by qPCR) per seed at site C for formulation F1. With formulation F2, *A. lipoferum* CRT1 was at 1.7 × 10^3^ CFU (on plates) and 3.8 × 10^5^ cell equivalents (by qPCR) per seed at sites L and FC, and 1.2 × 10^4^ CFU (on plates) and 1.2 × 10^6^ cell equivalents (by qPCR) per seed at site C.

### 4.2. Field Trials and Treatments

The field trials were run in 2015 in Chatonnay (site L), Sérézin-de-la-Tour (site FC) and Saint Savin (site C), which are located in the vicinity of Bourgoin-Jallieu (Isère, France). Details can be found in Rozier et al. [20]. The soils are classified (FAO system) as cambisol (C), luvisol (L) and fluvic-cambisol (FC), respectively. Each site grew wheat in 2013 and maize in 2014. The coated maize seeds were sown on April 30 (at C) and May 11 (at FC and L).

Each field experiment followed a factorial design with four combinations of factors, i.e., inoculation (*A. lipoferum* CRT1 or no inoculation) × formulation (standard formulation F2 or reduced formulation F1), and five randomised replicate blocks. The individual replicate plots (5 per treatment combination) were 12 rows (at FC and C) or 8 rows wide (at L) and 12 m long, and did not receive mineral nitrogen fertilizer.

### 4.3. Sampling of Maize

The maize was sampled at the six-leaf stage, on 27 May (C), 5 June (FC) and 8 June (L), as well as at the flowering stage, on 15 I, 16 (FC) and 17 (L) July. At each sampling, four root systems were taken per plot, i.e., 20 samples per treatment condition.

Upon uprooting, each root system was shaken to dislodge loosely adhering soil and flash-frozen in liquid nitrogen, followed by lyophilisation (24 h at −50 °C) in the laboratory. The root-adhering soil (i.e., rhizosphere soil) was separated from the roots using brushes and stored at −80 °C, prior to DNA extraction using the FastDNA SPIN kit (BIO 101 Inc., Carlsbad, CA, USA). The DNA samples (300 mg) were transferred to Lysing Matrix E tubes, along with 5 µL of the internal standard APA9 (at 10^9^ copies mL^−1^), in order to normalize the efficiency of the DNA extraction between the rhizosphere soil samples [34,56]. APA9 is vector pUC19 containing a cassava virus insert (GenBank accession number AJ427910), and was amplified with primers AV1f/AV1r, as described [34]. After a 1 h incubation at 4 °C, DNA was extracted and eluted in 50 mL sterile ultra-pure water, according to the manufacturer’s instructions, and DNA concentrations were assessed using Picogreen (ThermoFisher, Waltham, MA, USA).

### 4.4. Plant Growth Monitoring

Nine plant development parameters were measured on five six-leaves stage plants per plot (25 plants per treatment). The monitoring of root development was based on root dry biomass and root system architecture via the quantification of total root length, surface, and number, as well as average root diameter (WinRHIZO; Regent Instruments Inc., Québec City, Canada). The monitoring of shoot development was based on shoot dry biomass and stem diameter at the root collar, and on foliar morphology via the quantification of the length and average width of the fifth leaf from the shoot bottom (WinFOLIA; Regent Instruments Inc.). Data obtained with formulation F2 have been reported by [20,21].

### 4.5. Quantitative PCR Analysis of Microbial Functional Groups

The copy number of genes *nifH*, *acdS* and *phlD* in the maize rhizosphere was assessed by quantitative PCR using, respectively, (i) primers polF/polR [57] advocated for combined quantitative PCR and diversity analyses [45,58], (ii) primers acdSF5/acdSR8 validated for analysis of true *acdS* genes (i.e., without amplifying related D-cystein desulfhydrase genes coding for other types of PLP-dependent enzymes; [45,50]), and (iii) primers B2BF/B2BR3 [59]. Briefly, the reaction for *nifH* or *acdS* was performed in 20 µL containing 4 μL of PCR-grade water, 1 μL of each primer (final concentration 0.50 μM), 10 μL of LightCycler-DNA Master SYBR Green I master mix (Roche Applied Science, Meylan, France) and 2 µL of sample DNA (5 ng) [45]. The reaction for *phlD* was carried out in 20 µL containing 1.9 μL of PCR grade water, 1 μL of each primer (final concentration 1 μM), 10 μL of LightCycler-DNA Master SYBR Green I master mix (Roche Applied Science), 0.5 mg of T4g32 protein, 3% DMSO, and 2 µL of sample DNA (10 ng). PCR was performed for 10 min at 95 °C, followed by 50 cycles of: (i) 95 °C for 15 s (*nifH*), 94 °C for 15 s (*acdS*), or 94 °C for 30 s (*phlD*); (ii) 64 °C for 15 s (*nifH*), 67 °C for 15 s (*acdS*), or 67 °C for 7 s (*phlD*); and (iii) 72 °C for 10 s (*nifH* and *acdS*) or 15 s (*phlD*). A melting curve calculation and Tm determination were carried out using the Tm Calling Analysis module of Light-Cycler Software v.1.5 (Roche Applied Science). Real-time PCR quantification data were converted to gene copy number per gram of lyophilized root-adhering soil, as performed by [60]. Data obtained with formulation F1 have been shown [21].

### 4.6. Metabarcoding of rrs (Bacterial Community), nifH (Diazotrophs) and acdS (ACC Deaminase Producers)

Sequencing was carried out for samples from six-leaf maize. Equimolar composite samples of four rhizosphere DNA extracts (from four plants) were analysed per plot, i.e., 5 plots × 3 fields = 15 samples per treatment. Illumina MiSeq sequencing (2 × 300 bp for *nifH* and *rrs*; 2 × 125 bp for *acdS*) was performed by MR DNA laboratory (www.mrdnalab.com (accessed on 31 December 2021); Shallowater, TX, USA).

*nifH* and *acdS* sequencings were conducted using the same primers, polF/polR and acdSF5/acdSR8, respectively, whereas sequencing of the 16S rRNA gene *rrs* was conducted with primers 515/806 for the V4 variable region [61]. All forward primers carried a barcode. The 30-cycle PCR (5 cycles implemented on PCR products) was performed using the HotStarTaq Plus Master Mix Kit (Qiagen, Valencia, CA) with the following conditions: 94 °C for 3 min, followed by 28 cycles of 94 °C for 30 s, 53 °C for 40 s and 72 °C for 1 min, and a final elongation step at 72 °C for 5 min. The PCR products were checked in 2% agarose gel to verify the amplification success and relative band intensity. Multiple samples were pooled together in equal proportions based on their molecular weight and DNA concentrations. The pooled samples were purified using calibrated Ampure XP beads prior to preparing a DNA library following Illumina TruSeq DNA library preparation protocol. The reads have been deposited in the European Bioinformatics Institute (EBI) database under accession numbers PRJEB14346 (*nifH*), PRJEB14343 (*acdS*), PRJEB14347 (*rrs*).

The sequence data were processed using the analysis pipeline of MR DNA. Briefly, sequences were depleted of barcodes, the sequences < 150 bp or with ambiguous base calls were removed, the remaining sequences denoised, the operational taxonomic units (OTUs; defined at 3% divergence threshold for the three genes) generated, and the chimeras and singletons removed. Final the OTUs were taxonomically classified using BLASTn against a curated database derived from Greengenes [62], RDPII (http://rdp.cme.msu.edu) and NCBI (www.ncbi.nlm.nih.gov (accessed on 31 December 2021)). Final OTUs of the *acdS* sequencing were classified using an in-house curated *acdS* database [50].

*nifH* sequencing of six-leaf maize rhizosphere soil in 2015 gave 1,342,966 sequences (10,775 to 62,752 sequences per sample), corresponding to 36,241 OTUs. *acdS* sequencing resulted in 5,490,230 sequences (68,376 to 139,245 per sample), which yielded 32,468 OTUs. *rrs* sequencing was also performed to determine whether inoculation effects could also take place at the scale of the rhizobacterial community. A total of 6,082,255 reads were obtained (51,696 to 223,926 per sample), corresponding to 39,600 OTUs. For all three genes, rarefaction analysis indicated that the curves reached a plateau (Figures S1–S3). The relative abundance of bacterial genera identified following the analysis of *nifH, acdS* and *rrs* sequence reads is shown in Figures S4, S5 and S6, respectively. Data obtained with formulation F1 are shown in [21].

### 4.7. Statistical Analyses

The plant data and log-transformed quantitative PCR data were compared by one-factor ANOVA and Fishers’ LSD tests, or Kruskal–Wallis rank sum tests when data were non-parametric. The comparisons for bacterial composition data were carried out by Between-Class Analysis (BCA) [63,64], using ADE4 and ggplot2 packages for R (https://www.r-project.org/ 31 December 2021), and the Kruskal–Wallis rank sum tests associated to Tukey’s HSD tests. Non-metric multidimensional scaling (NMDS) was also performed, using the Bray–Curtis distance and the vegan package for R. The genus composition of the bacterial community and functional groups were characterized to consider the relative abundance of the most prevalent taxa. Statistical analyses were performed at *p* < 0.05, using R v3.1.2 (R Core Team [65]).

## Figures and Tables

**Table 1:** Effect of inoculum level of *Azospirillum lipoferum* CRT1 on root and shoot parameters of maize grown at the three fields sites L (Chatonnay), FC (Sérézin-la-Tour) and C (Saint Savin). Formulation F1 corresponds to the peat matrix used to coat seeds with low inoculant concentration, and the corresponding data in this table originate from [21]. In formulation F2, *A. lipoferum* CRT1 was used at higher inoculant concentration. Stem diameter was measured at root collar level and foliar morphology on leaf number five. At each field site, statistical differences between the four treatments are indicated by letters a–c (ANOVA and Fischer’s LSD tests, *p* < 0.05).

	Site L				Site FC				Site C			
Plant Parameters	Control		Inoculated		Control		Inoculated		Control		Inoculated	
	F1	F2	F1	F2	F1	F2	F1	F2	F1	F2	F1	F2
Shoot biomass (g plant^−1^)	0.34 ± 0.07 (b)	0.34 ± 0.07 (b)	0.43 ± 0.11 (a)	0.44 ± 0.1 (a)	0.52 ± 0.14 (bc)	0.50 ± 0.12 (c)	0.59 ± 0.17 (ab)	0.63 ± 0.15 (a)	0.34 ± 0.08 (b)	0.33 ± 0.07 (b)	0.39 ± 0.07 (a)	0.41 ± 0.08 (a)
Leaf length (cm plant^−1^)	13.1 ± 1.6 (b)	13.8 ± 1.6 (b)	14.0 ± 1.9 (b)	15.2 ± 1.7 (a)	20.7 ± 1.7 (a)	18.8 ± 1.9 (b)	19.8 ± 2.1 (ab)	20.6 ± 2.1 (a)	17.9 ± 2.4 (ab)	16.8 ± 1.7 (b)	17.2 ± 1.8 (ab)	18.3 ± 2.3 (a)
Leaf width (cm plant^−1^)	1.53 ± 0.15 (b)	1.61 ± 0.13 (ab)	1.68 ± 0.12 (a)	1.59 ± 0.17 (b)	1.61 ± 0.11 (a)	1.59 ± 0.12 (a)	1.64 ± 0.14 (a)	1.65 ± 0.14 (a)	1.71 ± 0.13 (a)	1.57 ± 0.11 (b)	1.71 ± 0.17 (a)	1.76 ± 0.14 (a)
Stem diameter (mm)	6.87 ± 0.73 (b)	6.95 ± 0.72 (b)	7.63 ± 0.78 (a)	7.82 ± 0.89 (a)	7.74 ± 1.07 (b)	7.55 ± 1.07 (b)	8.04 ± 0.99 (ab)	8.48 ± 0.94 (a)	7.24 ± 0.60 (ab)	6.83 ± 0.81 (b)	7.06 ± 0.84 (ab)	7.52 ± 0.79 (a)
Root biomass (g plant^−1^)	0.20 ± 0.05 (b)	0.23 ± 0.05 (ab)	0.24 ± 0.05 (a)	0.23 ± 0.05 (ab)	0.28 ± 0.05 (a)	0.26 ± 0.04 (ab)	0.23 ± 0.05 (b)	0.24 ± 0.05 (b)	0.27 ± 0.05 (a)	0.24 ± 0.06 (a)	0.25 ± 0.05 (a)	0.25 ± 0.05 (a)
Total root length (cm plant^−1^)	212 ± 60 (b)	225 ± 56 (b)	302 ± 86 (a)	330 ± 107 (a)	418 ± 109 (b)	498 ± 90 (a)	441 ± 90 (ab)	461 ± 65 (ab)	323 ± 86 (a)	280 ± 66 (a)	321 ± 61 (a)	278 ± 68 (a)
Total root surfaces (cm^2^ plant^−1^)	52 ± 12 (b)	58 ± 13 (b)	73 ± 20 (a)	72 ± 16 (a)	115 ± 29 (a)	124 ± 20 (a)	116 ± 28 (a)	121 ± 22 (a)	90 ± 24 (a)	79 ± 21 (ab)	82 ± 17 (ab)	71 ± 19 (b)
Average root diameter (mm)	0.79 ± 0.13 (a)	0.83 ± 0.10 (a)	0.78 ± 0.10 (ab)	0.71 ± 0.10 (b)	0.88 ± 0.08 (a)	0.80 ± 0.08 (b)	0.83 ± 0.08 (ab)	0.83 ± 0.07 (ab)	0.89 ± 0.07 (a)	0.89 ± 0.09 (a)	0.81 ± 0.08 (b)	0.81 ± 0.09 (b)
Number of roots	413 ± 120 (c)	793 ± 373 (b)	789 ± 469 (b)	2057 ± 1192 (a)	569 ± 87 (b)	595 ± 141 (b)	671 ± 250 (ab)	768 ± 273 (a)	804 ± 536 (a)	498 ± 213 (ab)	497 ± 139 (a)	390 ± 122 (b)

## Data Availability

Reads have been deposited in the European Bioinformatics Institute (EBI) database under accession numbers PRJEB14346 (*nifH)*, PRJEB14343 (*acdS*), PRJEB14347 (*rrs*).

## Supplementary Materials

The following supporting information can be downloaded at: https://www.mdpi.com/article/10.3390/microorganisms10020325/s1, Figure S1: Rarefaction curves for *rrs* at field sites L, FC and C. NI and I correspond, respectively, to non-inoculated and inoculated conditions. F1 and F2 correspond, respectively, to 10^4–5^ (reduced) and 10^5–6^ (standard) cells.seed^−1^ of the seed inoculant *A. lipoferum* CRT1. Data obtained with formulation F1 have already been shown in [21]; Figure S2: Rarefaction curves for *acdS* at field sites L, FC and C. NI and I correspond, respectively, to non-inoculated and inoculated conditions. F1 and F2 correspond, respectively, to 10^4–5^ (reduced) and 10^5–6^ (standard) cells.seed^−1^ of the seed inoculant *A. lipoferum* CRT1. Data obtained with formulation F1 have already been shown in [21]; Figure S3: Rarefaction curves for *nifH* at field sites L, FC and C. NI and I correspond respectively to non-inoculated and inoculated conditions. F1 and F2 correspond, respectively, to 10^4–5^ (reduced) and 10^5–6^ (standard) cells.seed^−1^ of the seed inoculant *A. lipoferum* CRT1. Data obtained with formulation F1 have already been shown in [21]; Figure S4: Relative abundance of bacterial genera based on analysis of *nifH* data pooled for field sites L, FC and C. NI and I correspond, respectively, to non-inoculated and inoculated conditions. F1 and F2 correspond, respectively, to 10^4–5^ (reduced) and 10^5–6^ (standard) cells.seed^−1^ of the seed inoculant *A. lipoferum* CRT1. Data obtained with formulation F1 have already been shown in [21]; Figure S5: Relative abundance of bacterial genera based on analysis of *acdS* data pooled for field sites L, FC and C. NI and I correspond, respectively, to non-inoculated and inoculated conditions. F1 and F2 correspond, respectively, to 10^4–5^ (reduced) and 10^5–6^ (standard) cells.seed^−1^ of the seed inoculant *A. lipoferum* CRT1. Data obtained with formulation F1 have already been shown in [21]; Figure S6: Relative abundance of bacterial genera based on analysis of *rrs* data pooled for field sites L, FC and C. NI and I correspond, respectively, to non-inoculated and inoculated conditions. F1 and F2 correspond, respectively, to 10^4–5^ (reduced) and 10^5–6^ (standard) cells.seed^−1^ of the seed inoculant *A. lipoferum* CRT1. Data obtained with formulation F1 have already been shown in [21]; Figure S7: Non-metric multidimensional scaling (NMDS) of *rrs* data at all three field sites L (Chatonnay), FC (Sérézin-de-la-Tour) and C (Saint Savin) (A), at field site L only (B), at field site FC only (C), and at field site C only (D). NI and I correspond, respectively, to non-inoculated and inoculated conditions. F1 and F2 correspond, respectively, to 10^4–5^ (reduced) and 10^5–6^ (standard) cells.seed^−1^ of the seed inoculant *A. lipoferum* CRT1. NMDS of distance matrices was based on the Bray–Curtis distance and was done using the vegan package in R; Figure S8: Non-metric multidimensional scaling (NMDS) of *nifH* data at all three field sites L (Chatonnay), FC (Sérézin-de-la-Tour) and C (Saint Savin) (A), at field site L only (B), at field site FC only (C), and at field site C only (D). NI and I correspond, respectively, to non-inoculated and inoculated conditions. F1 and F2 correspond, respectively, to 10^4–5^ (reduced) and 10^5–6^ (standard) cells.seed^−1^ of the seed inoculant *A. lipoferum* CRT1. NMDS of distance matrices was based on the Bray–Curtis distance and was done using the vegan package in R; Figure S9: Non-metric multidimensional scaling (NMDS) of *acdS* data at all three field sites L (Chatonnay), FC (Sérézin-la-Tour) and C (Saint Savin) (A), at field site L only (B), at field site FC only (C), and at field site C only (D). NI and I correspond, respectively, to non-inoculated and inoculated conditions. F1 and F2 correspond, respectively, to 10^4–5^ (reduced) and 10^5–6^ (standard) cells.seed^−1^ of the seed inoculant *A. lipoferum* CRT1. NMDS of distance matrices was based on the Bray–Curtis distance and was done using the vegan package in R.
