# Supplementary material for: Effect of Inoculation Level on the Impact of the PGPR Azospirillum lipoferum CRT1 on Selected Microbial Functional Groups in the Rhizosphere of Field Maize
Source: Microorganisms. 2022 Jan 31;10(2):325. doi: 10.3390/microorganisms10020325 (PMC8877547; doi:10.3390/microorganisms10020325)
Supplement: Supplementary file 1 [file microorganisms-10-00325-s001.zip › microorganisms-1560242-supplementary/microorganisms-1560242-supplementary.pptx]

## Slide 1
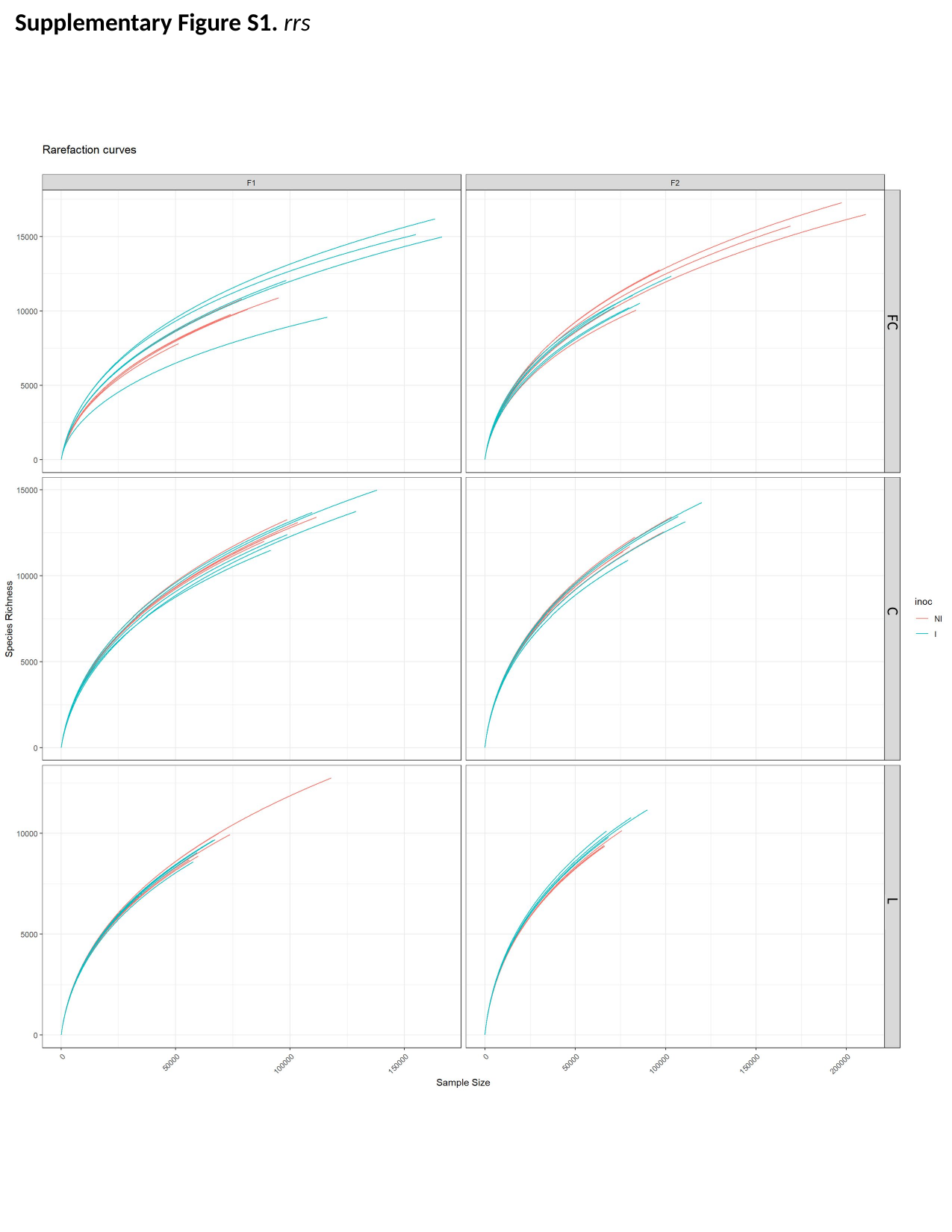

Supplementary Figure S1. rrs
FC
C
L

## Slide 2
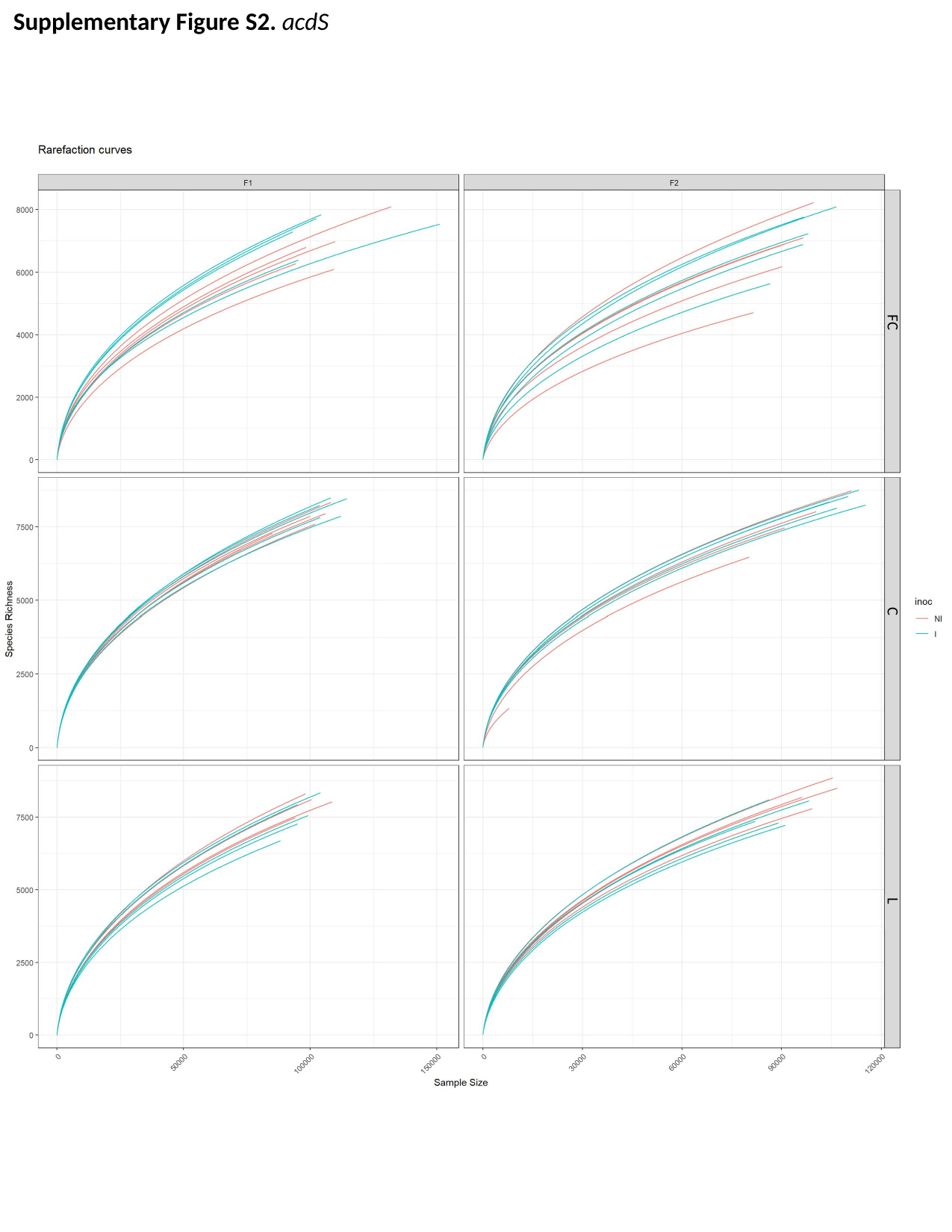

Supplementary Figure S2. acdS
FC
C
L

## Slide 3
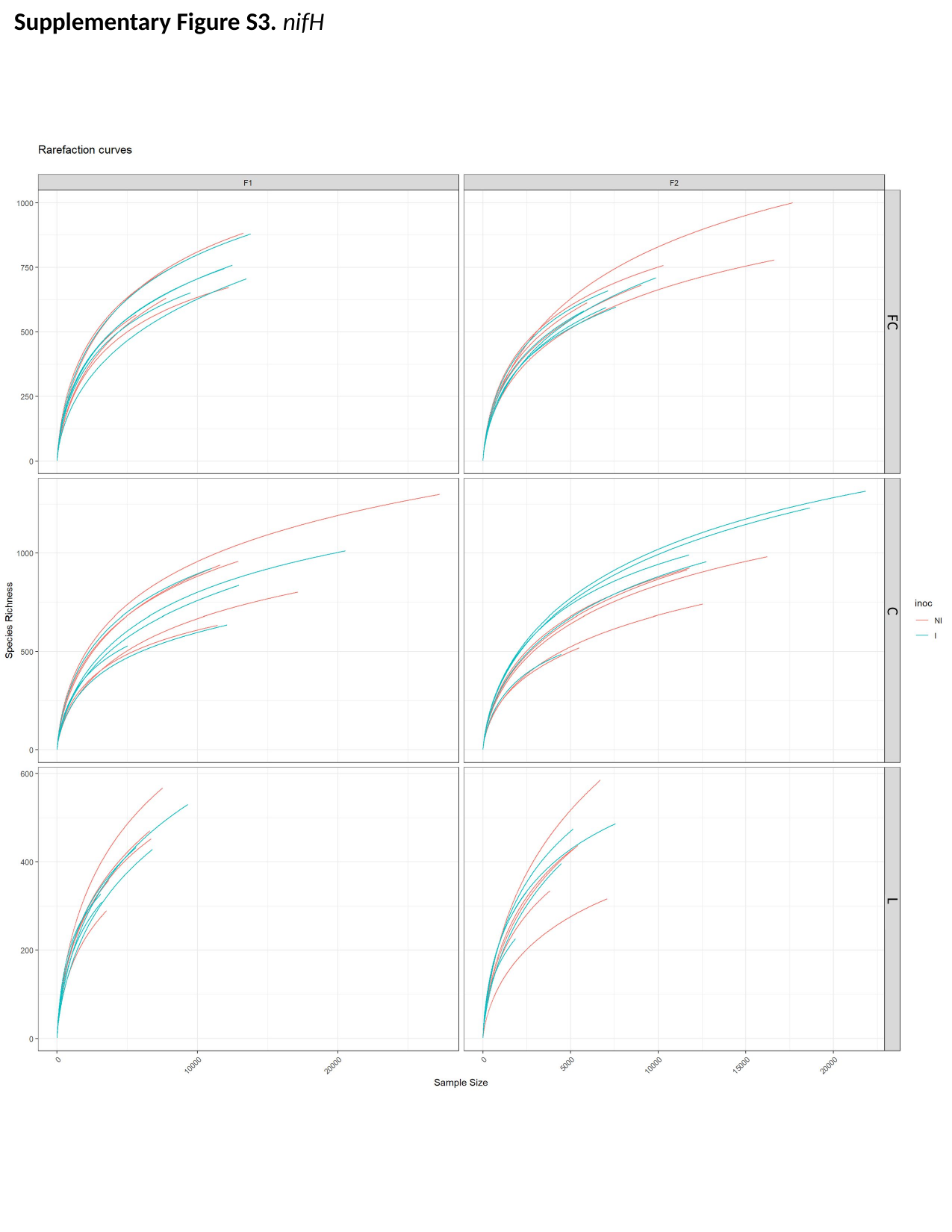

Supplementary Figure S3. nifH
FC
C
L

## Slide 4
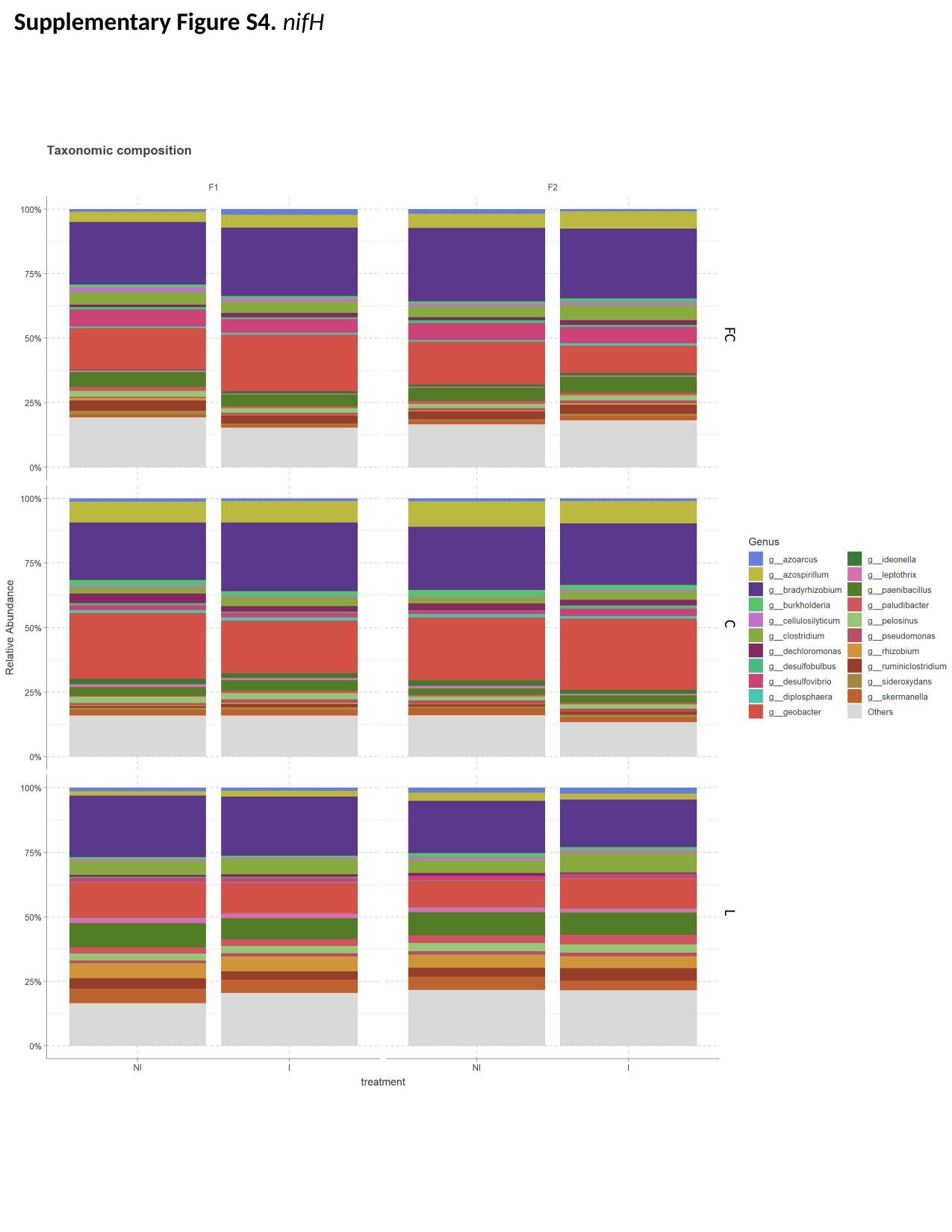

Supplementary Figure S4. nifH
FC
C
L

## Slide 5
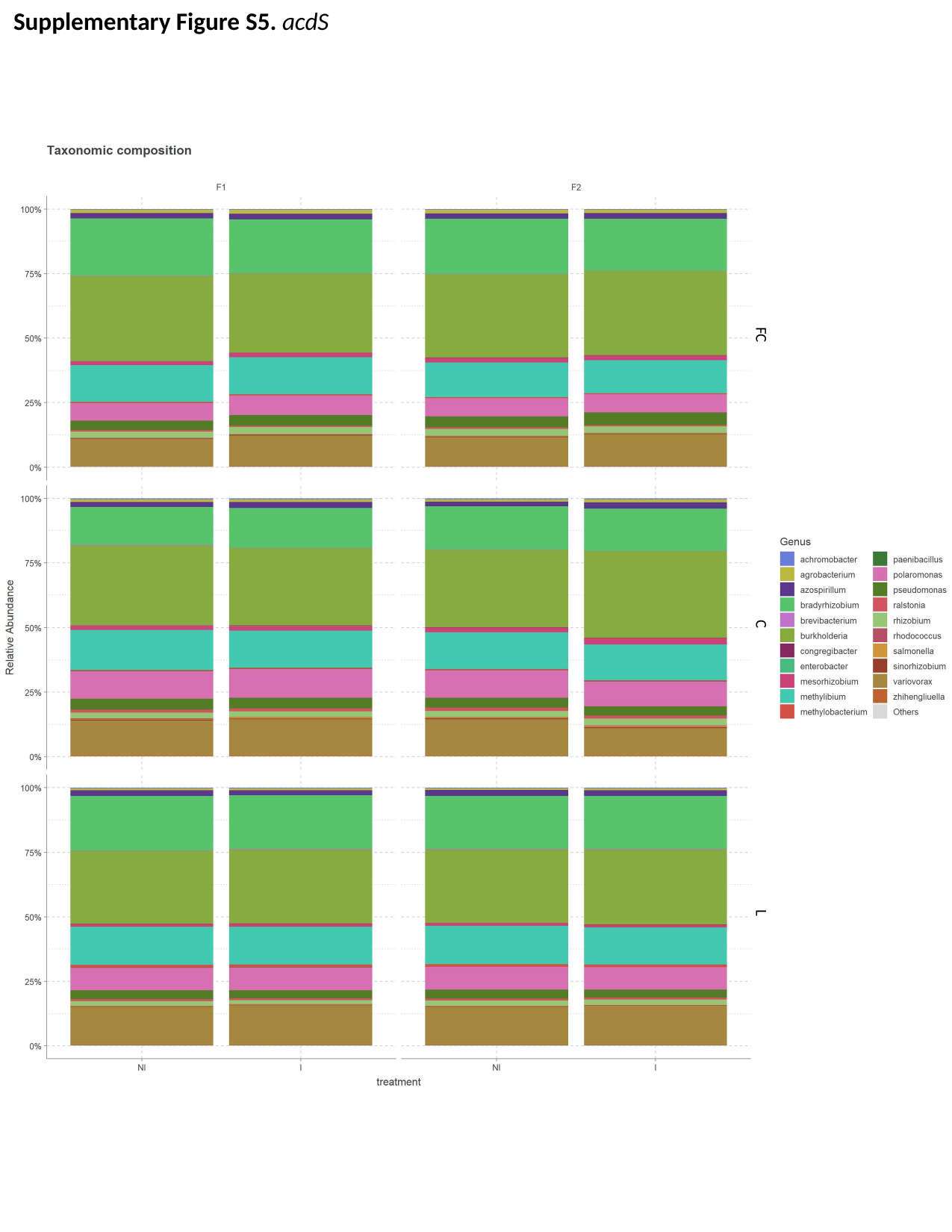

Supplementary Figure S5. acdS
FC
C
L

## Slide 6
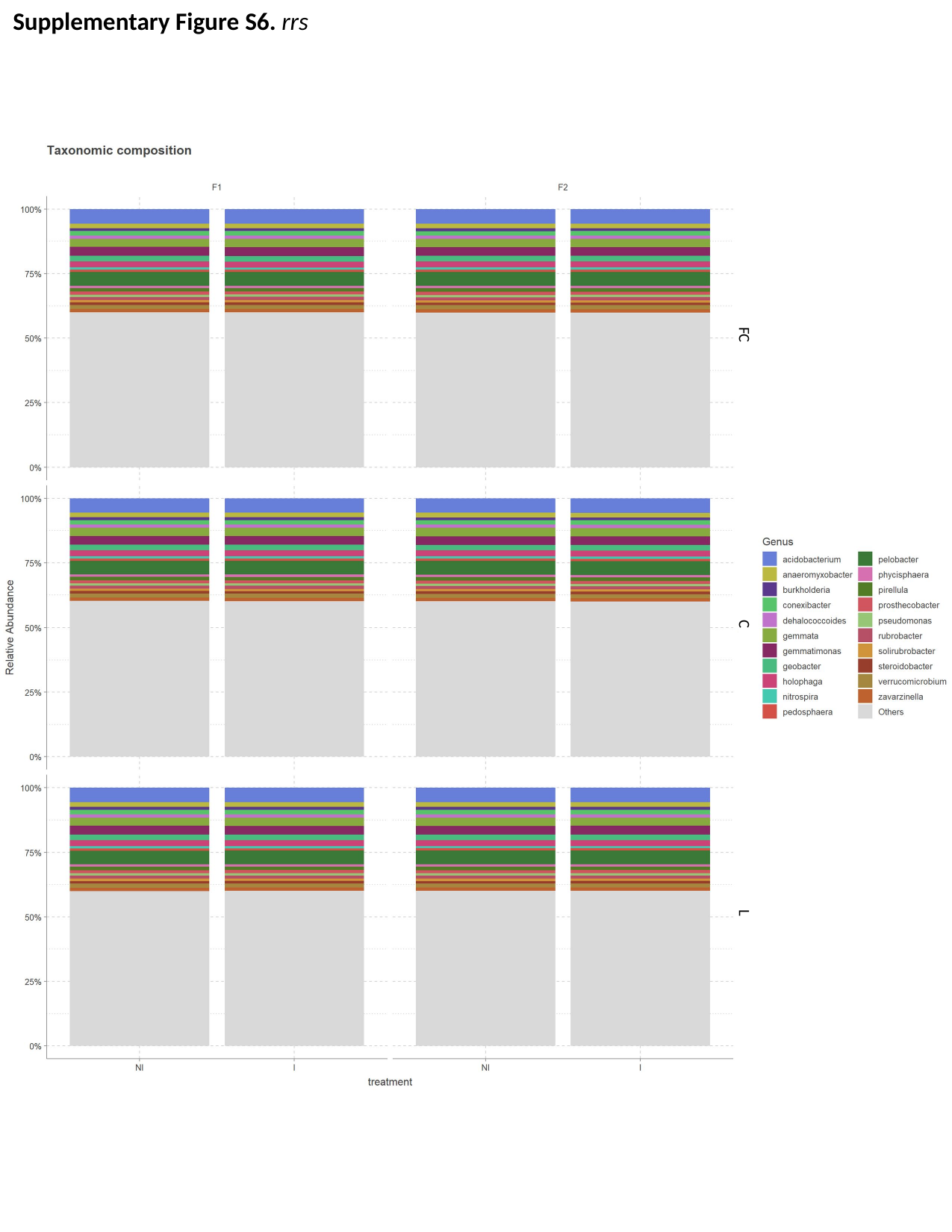

Supplementary Figure S6. rrs
FC
C
L

## Slide 7
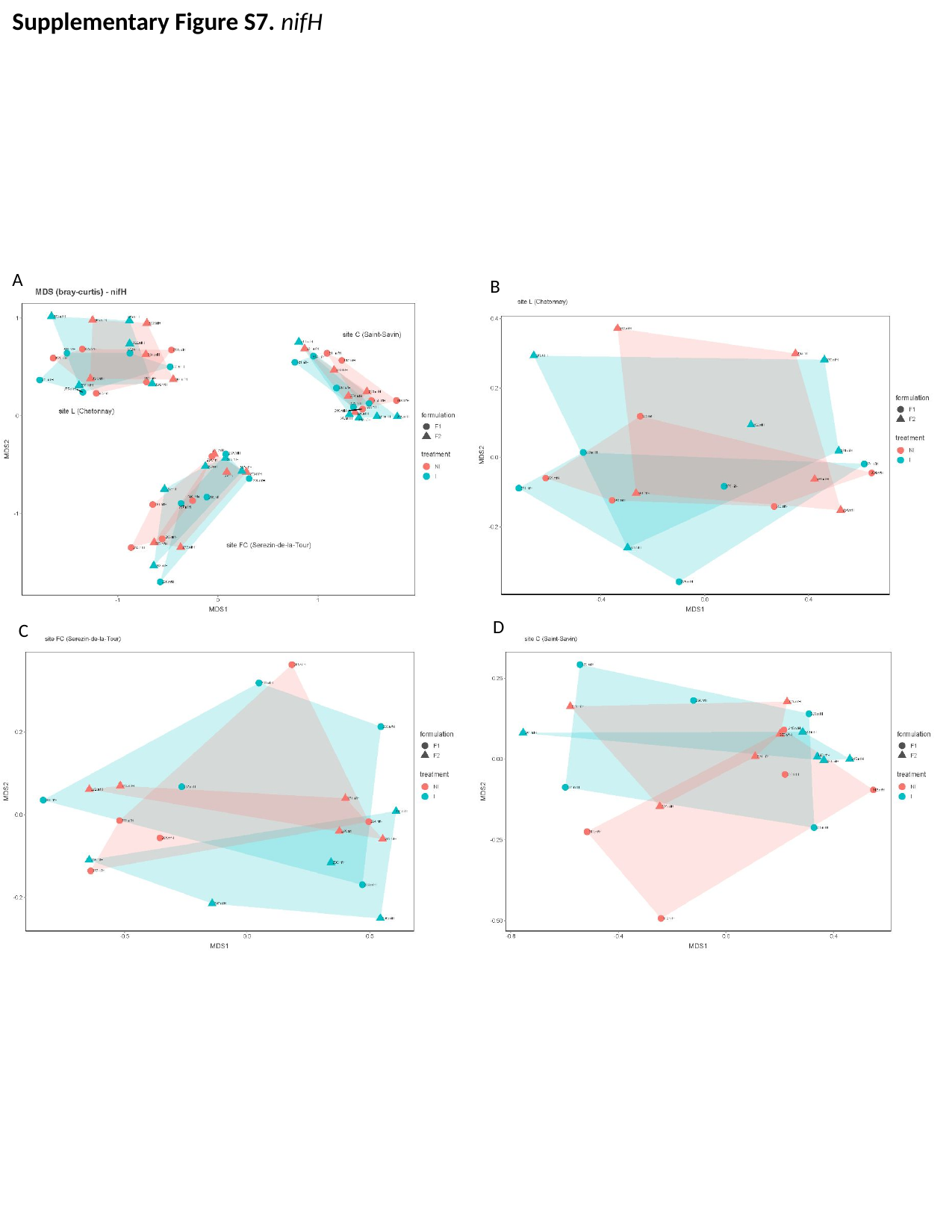

Supplementary Figure S7. nifH
A
B
D
C

## Slide 8
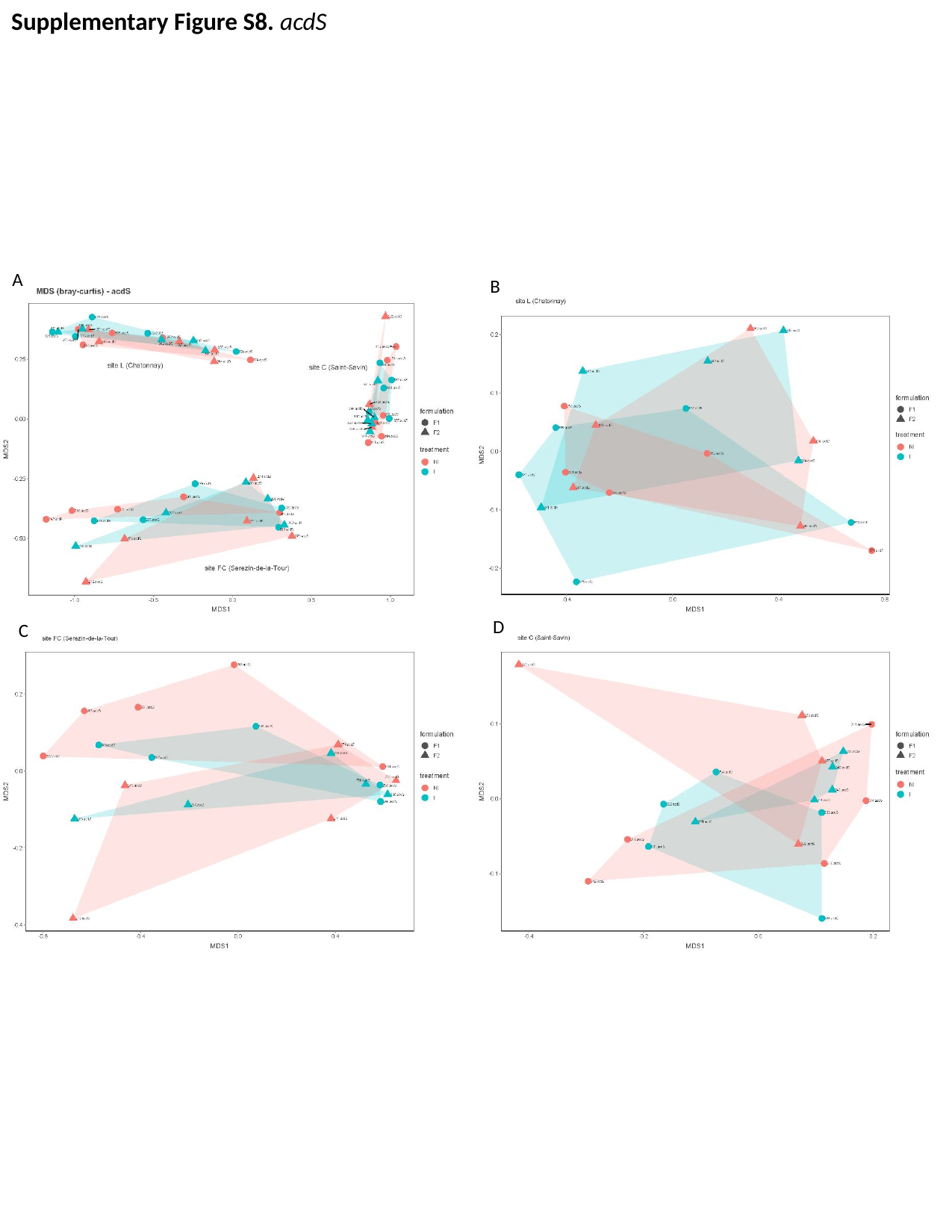

Supplementary Figure S8. acdS
A
B
D
C

## Slide 9
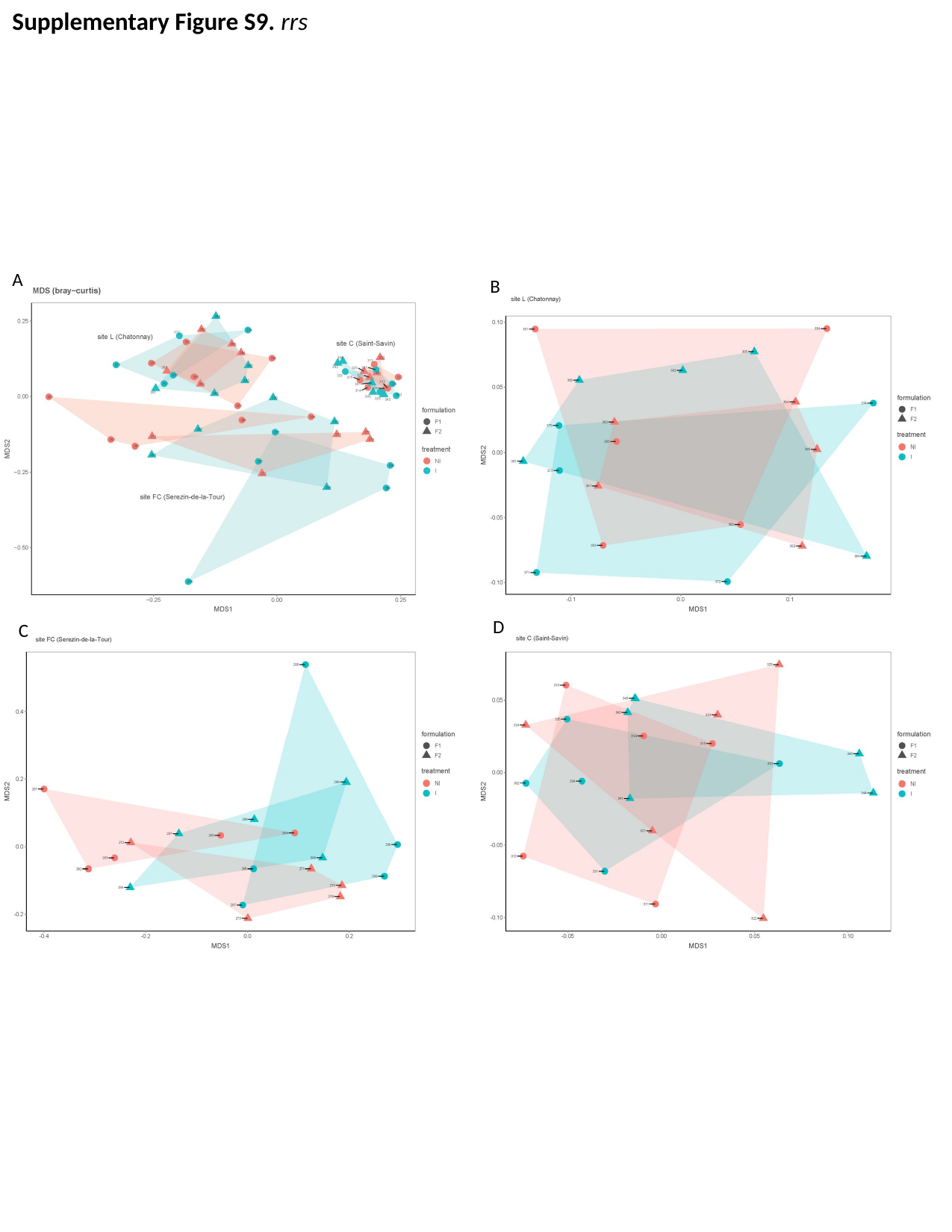

Supplementary Figure S9. rrs
A
B
D
C
